# Magnetic Resonance Imaging (MRI) and MR Spectroscopic Methods in Understanding Breast Cancer Biology and Metabolism

**DOI:** 10.3390/metabo12040295

**Published:** 2022-03-27

**Authors:** Uma Sharma, Naranamangalam R. Jagannathan

**Affiliations:** 1 Department of NMR & MRI Facility, All India Institute of Medical Sciences, New Delhi 110 029, India; 2 Department of Radiology, Chettinad Hospital & Research Institute, Chettinad Academy of Research & Education, Chennai 603 103, India; 3 Department of Radiology, Sri Ramachandra Institute of Higher Education and Research, Chennai 600 116, India; 4 Department of Electrical Engineering, Indian Institute of Technology (IIT) Madras, Chennai 600 036, India

**Keywords:** breast cancer, biology, metabolism, metabolomics, nuclear magnetic resonance (NMR), magnetic resonance imaging (MRI), magnetic resonance spectroscopy (MRS), biomarkers, therapeutic response

## Abstract

A common malignancy that affects women is breast cancer. It is the second leading cause of cancer-related death among women. Metabolic reprogramming occurs during cancer growth, invasion, and metastases. Functional magnetic resonance (MR) methods comprising an array of techniques have shown potential for illustrating physiological and molecular processes changes before anatomical manifestations on conventional MR imaging. Among these, in vivo proton (^1^H) MR spectroscopy (MRS) is widely used for differentiating breast malignancy from benign diseases by measuring elevated choline-containing compounds. Further, the use of hyperpolarized ^13^C and ^31^P MRS enhanced the understanding of glucose and phospholipid metabolism. The metabolic profiling of an array of biological specimens (intact tissues, tissue extracts, and various biofluids such as blood, urine, nipple aspirates, and fine needle aspirates) can also be investigated through in vitro high-resolution NMR spectroscopy and high-resolution magic angle spectroscopy (HRMAS). Such studies can provide information on more metabolites than what is seen by in vivo MRS, thus providing a deeper insight into cancer biology and metabolism. The analysis of a large number of NMR spectral data sets through multivariate statistical methods classified the tumor sub-types. It showed enormous potential in the development of new therapeutic approaches. Recently, multiparametric MRI approaches were found to be helpful in elucidating the pathophysiology of cancer by quantifying structural, vasculature, diffusion, perfusion, and metabolic abnormalities in vivo. This review focuses on the applications of NMR, MRS, and MRI methods in understanding breast cancer biology and in the diagnosis and therapeutic monitoring of breast cancer.

## 1. Introduction

Breast cancer is a significant healthcare challenge and a major reason for cancer-related mortality among women all over the World [1]. Early detection and therapy contribute to the survival increase and clinical outcomes of breast cancer patients. Despite significant advancement directed towards improving diagnostic and therapeutic approaches, early diagnosis and therapeutic response/resistance remain a clinical challenge. Breast tumors exhibit considerable heterogeneity, which contributes to varying therapeutic responses. This heterogeneous nature in terms of hormonal receptor status and human epidermal growth factor receptor 2 (HER2) amplification is used to guide targeted therapy. A malignant cell acquires distinct characteristics of unlimited replication potential, angiogenesis, tissue invasion, metastases, resistance to apoptosis, and metabolic reprogramming, which support the formation of a tumor mass and its growth [2]. Thus, a comprehensive understanding of the underlying biochemical, vascular and functional properties contributing to tumor growth may help develop better diagnostic/monitoring and therapeutic approaches.

The tools based on the magnetic resonance (MR) phenomenon offer various distinct features of breast tumors that were explored for effective clinical management during the last three decades. Multi-parametric MR-based approaches showed the potential to classify patients according to pathology or their responses to treatment and improve clinical outcomes. Dynamic contrast-enhanced magnetic resonance imaging (DCE-MRI) is the standard technique for breast imaging which relies on the administration of contrast agents and reflects the tumor vascularity, morphology, and kinetics of breast lesions [3]. DCE-MRI is established as a screening modality for women with various risk profiles, the sensitivity ranges (81–100%) [4]. Tumor proliferation requires the generation of new vessels or angiogenesis for the supply of nutrients to cells. These vessels differ in characteristics from normal vessels as they have larger diameters, lack contractile properties, and have more permeability [5]. The characteristics of tumor vessels are measured by perfusion-weighted imaging (PWI), and it has become a promising tool for characterizing tumor pathophysiology [6].

Diffusion-weighted imaging (DWI), which measures the motion of water molecules in tissues, is sensitive to cell density, microstructure, and membrane integrity. For example, studies documented that malignant breast lesions showed decreased water diffusion, attributed primarily to the increased cellularity, enabling the differentiation of malignant and benign breast tumors [7,8,9,10,11,12,13,14]. Furthermore, the viscoelastic properties of tissues can be quantitated using MR elastography (MRE) [15,16,17].

The cancer cells also reprogram their metabolic pathways to fulfill the continuous supply of materials required for the biosynthesis of membranes, genes, and proteins [18]. Magnetic resonance spectroscopy (MRS) is an important tool that is used majorly in three forms, in vivo, ex vivo, and in vitro, to characterize the metabolic state of malignant, benign, and normal breast tissues. The potential of using in vitro nuclear magnetic resonance (NMR)-based metabolic profiling of tissue extracts, cell lines, and biofluids is reported to identify a large number of small molecules as potential biomarkers for diagnosis and therapy monitoring [19,20,21,22,23,24,25,26,27,28,29,30,31]. Studies also used solid-state MR spectroscopic analysis of intact biopsied tissues using the high-resolution magic angle spinning (HRMAS) method to monitor metabolite levels for the diagnosis/prognosis of breast tumors [32,33,34,35,36,37,38,39]. Breast in vivo MRS studies showed high levels of choline-containing metabolites (tCho), indicating the rapid proliferation of malignant tumors [40,41,42,43,44,45,46,47]. Recently, hyperpolarized ^13^C MRI (HP ^13^C MRI) was also explored to probe the altered tumor metabolism [48].

**Figure 1:**
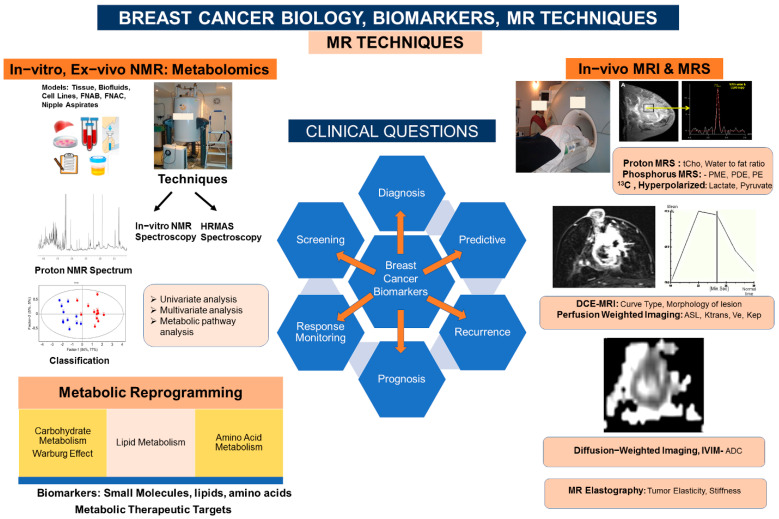
Schematic representation of various MRI and MRS techniques and biomarkers obtained in studying breast cancer biology and metabolism.

This review briefly describes the potential of various MRI and MRS methods in studying breast cancer biology and metabolism and their role in determining biomarkers for diagnosis and therapeutic monitoring (Figure 1). Table 1 compare the advantages and limitations of MRS and MRI studies.

## 2. Breast Cancer Biology: Metabolic Reprogramming

The altered composition of metabolites in disease states such as cancer helps provide meaningful information on the associated metabolic reprogramming of cancer progression. The following section briefly presents the importance of altered metabolites in understanding the metabolic reprogramming associated with tumorigenesis in breast cancer.

Glucose (Glc) is the primary energy source in normal cells, which is converted into pyruvate through the glycolysis pathway. Under normal conditions, pyruvate is converted to acetyl co-enzyme A which enters into the tricarboxylic acid cycle for further reactions of energy generation. Nicotinamide adenine dinucleotide (NADH) and flavine adenine dinucleotide (FADH2) molecules are formed through the tricarboxylic acid cycle oxidized through oxidative phosphorylation, also known as the electron transport pathway to produce adenosine triphosphate molecules, which serve as the energy currency of cells. In anaerobic conditions, pyruvate (Pyr) is dehydrogenated to lactate (Lac) for energy generation, which is less energy-efficient than oxidative phosphorylation. Most NMR studies reported higher lactate levels in breast cancers, indicating a higher rate of glycolysis despite the process being less energy efficient.

Interestingly, even with sufficient oxygen levels, tumor cells have higher rates of glycolysis than normal cells; this condition is referred to as aerobic glycolysis [49,50]. The alterations in enzyme regulation mechanisms occur in tumors which supports the higher rate of glycolysis. Low levels of ATP activate enzyme phosphofructokinase, which leads to higher levels of fructose 1,6 di-phosphate and consequently a higher level of Pyr [51]. In addition, an increased concentration of fructose 1,6 di-phosphate is documented in tumors, leading to the significant activation of pyruvate kinase and enhanced Lac production [51].

Higher rates of glycolysis are an adaptation of the metabolism to facilitate the production of the substrates needed for rapid proliferation [52]. For example, ribose-phosphate produced by the pentose phosphate pathway is required for nucleic acid synthesis. Additionally, a higher level of Lac is favorable for tumors, making them resistant against the immune system and more destructive for the surrounding tissue [53]. Inefficient ATP production is compensated by an increased rate of Glc uptake in tumors. Haukaas et al. reported three metabolic clusters of breast cancer that showed differences in protein as well as in breast cancer-related genes, indicating that the molecular heterogeneity of tumors is also found to express at the metabolic level [54].

The HRMAS MRS analysis of 228 tumor samples revealed differences in the metabolic profiles, which could be categorized into three different metabolic clusters (Mc1, Mc2, and Mc3) based on combining metabolic profiling with gene expression and protein expression profiles. The Mc1 showed the highest levels of membrane metabolites GPC and PC. Mc2 was characterized with the highest levels of Glc, while Mc3 showed the most elevated levels of alanine and lactate [54].

**Figure 2:**
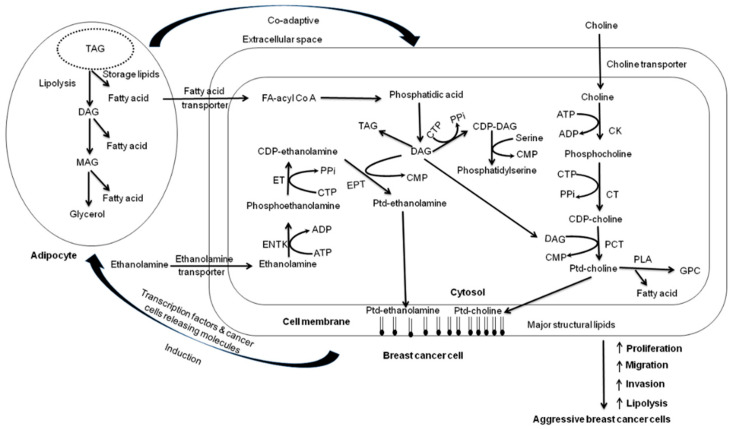
Metabolic reprogramming in breast cancer cells, its role, and the induced co-adaptive mechanism (Reproduced with permission from John Wiley & Sons, Inc. from Reference [44]).

Breast cancer also showed abnormalities in choline and lipid metabolism [55] and significantly higher levels of choline-containing compounds (tCho), especially phosphocholine (PC), compared to normal tissue/cells [19,20,21,22,23,24,25,26,27,28,29,30,31,32,33,34,35,36,37,38,39,40,41,42,43,44,45,46,47,56,57,58,59]. Cell culture studies documented the association of PC levels with rapid proliferation [60,61]. A correlation between the PC level and the proliferative state in cell culture was reported; low PC levels were found in nonproliferative cells [60]. Phospholipids phosphatidylcholine (PtdCho) and phosphatidylethanolamine (PtdEtn) are major constituents of cell membranes. An understanding of the biosynthesis of PtdCho explains the increased levels of these metabolites in rapidly dividing cells. Glycerophosphocholine (GPC) and PC are important metabolites of phospholipid metabolism. The biosynthesis of PtdCho takes place via a three-step pathway, also known as the Kennady Pathway. It is regulated by three enzymes, namely choline kinase (CK), phosphocholine transferase (PCT), and CTP-cytidyl transferase (CT). The metabolites PC and phosphoethanolamine (PE) serve as precursors for the synthesis of PtdCho and PtdEtn. PC is produced by the phosphorylation of Cho, and this reaction is catalyzed by the enzyme CK. Stimulation factors such as hormones, growth factors, fetal serum, or tumor promoters induce the activation of the enzyme CK. It increases the phosphorylation of choline to PC, consequently increasing its level and biosynthesis of PtdCho [62] (see Figure 2).

Further, the membrane phospholipids PtdCho and PtdEtn are hydrolyzed by enzyme phospholipase, and PC, GPC, and PE are produced. Thus these compounds play the role of a precursor and as a product in the phospholipid metabolism. It is known that increased membrane turnover requires the rapid synthesis and degradation of phospholipids in tumor cells. The hydrolysis of PtdCho is mediated by three kinds of phospholipases, specifically phospholipases, PLA2, PLC, and PLD. The phospholipases play a dual role in balancing the degradation and the synthesis of phospholipids. Breast cancer cell lines show elevated expression of PLD compared to normal cell lines [63]. Further, increased PLC activity and CK activity are also reported in breast cancer cells [64]. Katz-Brull et al. reported a faster rate of transport of Cho and its phosphorylation in MCF-7 breast cancer cell lines than that seen in normal epithelial mammary cells [61]. These results were also supported by a reduction in choline transport in response to TNF therapy in human breast cancer cells [65]. These studies suggested that both the increased transportation and the enhanced CK and PLD activities contributed to the increased levels of PC in malignant cells.

The molecular heterogeneity of breast tumors is also manifested as higher tCho levels, indicating that there may be changes in the phospholipid metabolism in tumors of various molecular subtypes. An in vivo MRS study by Tozaki and Hoshi showed a correlation of tCho with ER status, triple-negative (TN) status, and nuclear grade [66]. Choline levels correlate with the expression of calcium-sensing receptors, suggesting its role in choline synthesis in breast cancer [67]. Patients with TN status had significantly lower tCho than those with non-TN and triple positive cases [42]. Recently, we reported the association of tCho with the Wnt/β-catenin pathway in malignant breast lesions [68]. In malignant tissues, tCho showed a positive correlation with nuclear and cytosolic expressions of β-catenin and cyclin D1. Higher cytosolic β-catenin expression was found in PR negative patients than PR positive [68].

Additionally, breast tumors modulate amino acid metabolism. In tumor cells, glutamate (Glu) and glutamine (Gln) are also utilized as an energy source by entering into the TCA cycle [69,70]. Glutamate is also used for the synthesis of glutathione, an important antioxidant, or its amino group can also be used for the synthesis of nonessential amino acids such as aspartate, alanine (Ala), glycine (Gly), and serine [69,70]. Glycine can be synthesized from its precursor serine from 3-phosphoglycerate, an intermediate of glycolysis. It can also be synthesized by the oxidation of Cho to betaine. Betaine is then converted to sarcosine, which is converted to Gly. It is reported that mitochondrial serine hydroxymethyltransferase 2 (SHMT2) is overexpressed in human tumors. It catalyzes the conversion of serine to Gly [71]. High Gly is found to be associated with poor prognosis [37,72]. Thus, altered levels of the above amino acids suggests the presence of adapting metabolic pathways that support tumor growth. Further, elevated levels of the amino acid taurine (Tau) are found in breast cancer [33,72,73]. Lower Tau levels are seen in ER- compared to ER+ and in HER2+ compared to negative tumors [73].

## 3. Breast Biomarkers: NMR Based Metabolomics, Metabolic Fingerprinting

Metabolomics is a holistic study of the chemical fingerprints of metabolites or small molecules in tissues, biofluids, or organisms [74,75,76,77,78]. These metabolic fingerprints are related to various metabolic processes and environmental alterations. It comprehensively quantifies and analyzes exogenous and endogenous metabolites of the metabolome with high throughput to discover new diagnostic biomarkers of diseases. Many metabolites, including sugars, amino acids, organic acids, lipids, fatty acids, and numerous other small molecules, provide holistic information on the metabolic and physiological state, offering new insight into pathogenesis and treatment strategies [74,75,76,77,78]. Metabolic fingerprinting using a high-throughput tool such as NMR spectroscopy has become a powerful system biology approach to discover biomarkers and understand complex disease processes. The group of Nicholson developed statistical approaches combining NMR methods for the noninvasive rapid characterization of metabolic fingerprints [74,75,76,77,78]. Garcia-Perez et al. recently described a system for identifying molecules in NMR-based metabolic phenotyping, including information on sample preparation, spectral acquisition, and statistical modeling. The multi-platform system proposed to identify signals in the NMR spectra corresponding to the same molecule using statistical total correlation spectroscopy (STOCSY), subset optimization by reference matching (STORM), and resolution-enhanced (RED)-STORM [74]. Spectral databases listing the metabolites present in biofluids such as urine and blood are available. NMR offers an array of experiments that can be used according to the nature of the sample. Analyzing the metabolic profile of a biological specimen, elucidating metabolite structure, and metabolite detection in living tissue are all possible using NMR. However, different hardware and detection pulse schemes are required for in vivo and in vitro NMR measurements. For in vitro and ex vivo metabolic fingerprinting, an array of NMR experiments such as one-dimensional (1D), two-dimensional (2D), and higher dimensional homo- and hetero-nuclear can be performed for comprehensive metabolic profiling studies [74,79]. Proton (^1^H) is the most sensitive and abundant nuclei present, and hence, it is commonly used for NMR-based metabolomics studies. However, other nuclei such as ^13^C, ^31^P can be used for specific applications. Readers are referred to the literature for a more detailed description of the various NMR techniques and protocols employed for metabolomics study [74,79].

### 3.1. Tumor Tissue, Axillary Nodes: HRMAS and In Vitro MRS Studies

High-resolution magic angle spinning (HRMAS) MRS emerged as a valuable tool for studying the metabolomics of intact tumor tissues [32,33,34]. Sitter et al. reported a correlation of metabolite biomarkers such as Lac, PC, and lipids with the histopathological grade [33]. Li et al. showed elevated levels of Cho-containing compounds and Tau in cancer compared to noncancer tissue [80]. Elevated PC was suggested as a potential biomarker in identifying the resection margin [81]. Gogiashvili et al. reported that considerable metabolic heterogeneity exists within a tumor [82]. The pure DCIS lesions were differentiated from DCIS with invasive carcinoma using a higher GPC/PC ratio, myo-inositol, and succinate [83]. The metabolites PC, Cho, and Gly were found at high levels in tumors with a high signal enhancement ratio and high SUV by PET-CT [84]. Metabolic data were correlated with the gene expression for refining the sub-classification of breast cancers [85]. MR profiles predicted important prognostic factors such as ER and PR and axillary node status, benefitting treatment planning [35,36,86,87]. A correlation among gene, protein expression, and metabolic profiles documented that breast tumors exhibited three different metabolic clusters [54]. Additionally, TN cancer had a lower Gln level than triple-positive breast cancers, documenting increased glutaminolysis in the TN group and suggesting it as a new therapeutic target [87]. Choi et al. reported that breast cancer patients with pathologic complete response showed lower tCho and PC/Cr ratio levels than patients with no complete pathologic response to neoadjuvant chemotherapy [88].

**Figure 3:**
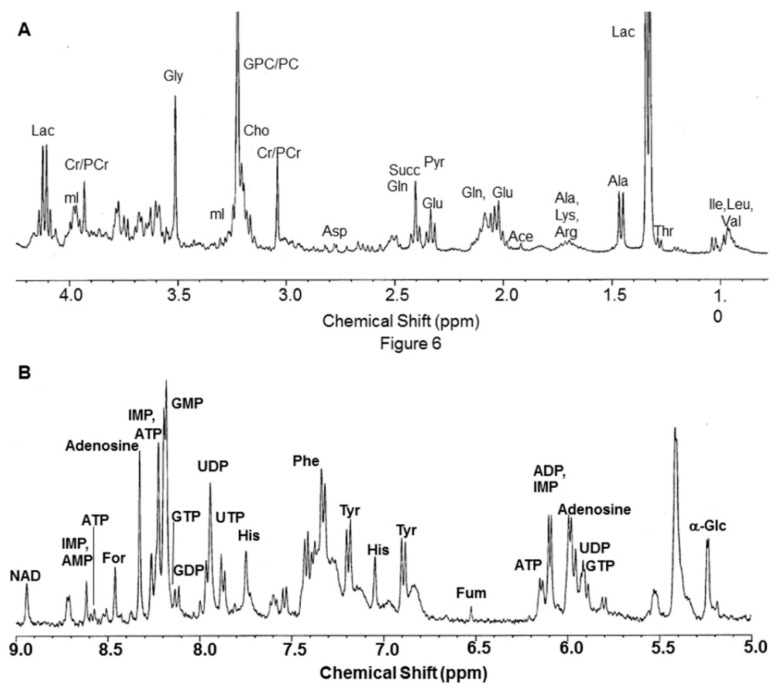
(**A**) 1D ^1^H NMR spectrum region showing the metabolite resonances from 0.8 to 4.2 ppm recorded at 400 MHz of perchloric acid extract (pH 7) of involved axillary lymph node of a breast cancer patient. Pyr = pyruvate; Arg = arginine; Gly = glycine. (**B**) The expanded region showing the metabolite resonances from 5 to 9 ppm of the same patient. NAD = nicotinamide adenine dinucleotide; IMP = inosine monophosphate; GMP = guanosine monophosphate; GTP = guanosine triphosphate; GDP = guanosine diphosphate; UDP = uridine diphosphate; Tyr = tyrosine (Reproduced with permission from Elsevier from Reference [24]).

Few studies explored the metabolic profiling of tumor extracts and axillary nodes using in vitro MRS [19,21,24,89,90]. In an initial study, Gribbestad et al. reported the metabolic profiling of breast cancer tissue extracts, showing significant differences in the metabolite levels between involved (cancerous tissue) and noninvolved (normal breast tissue from surrounding areas) breast tissues [19]. Significantly increased concentrations of Ala, lysine, glutamic acid, Gln, Lac, acetate, phosphocreatine+creatine, myo-inositol, Cho, and GPC + PC were reported in cancerous breast tissue compared to non-involved tissues, suggesting altered metabolism in cancer tissues [21]. Variations in the levels of PC, PE, and uridine di-phosphate-hexose were related to tumor grade [89]. The potential of in vitro NMR in breast cancer prognosis was examined by the metabolic status of metastatic and noninvolved lymph nodes [24,90]. Lac, Ala, GPC + PC, Cho, and uridine-di-phosphoglucose were significantly higher in nodes with metastases (Figure 3) [24]. Using a ratio of metabolites [(GPC + PC)/Threonine], as a biomarker, axillary node metastases were detected with 80% sensitivity, 91% specificity, and 88% accuracy [84].

### 3.2. Biofluids

Several studies examined the potential of the ^1^H NMR-based metabolomics of blood plasma in understanding disease progression by evaluating the metabolome of early and late-stage breast cancers [28,29,91,92]. Jobard et al. identified nine statistically significant metabolites involved in the discrimination of early breast cancer (EBC) and metastatic breast cancer (MBC): histidine, Glu, phenylalanine acetoacetate, Pyr, glycerol, glycoproteins (N-acetyl), and mannose [28]. Sixteen metabolites, including lysine, Glu, hydroxybutyrate, Glc, Lac, and N-acetyl glycoprotein, showed significant differences between EBC and MBC [29]. Similarly, the comparison of the metabolome of the filtered plasma of EBC and MBC patients reported that Lac showed an inverse correlation with the tumor size in EBC [91]. The potential of the plasma metabolomics approach was also evaluated for detecting micro-metastatic disease in patients with EBC to improve risk stratification [92,93]. Asiago et al. reported the same using the metabolic profiling of serum by NMR and GCMS. Eleven metabolites could be identified as biomarkers for predicting breast cancer recurrence [94].

The use of plasma/sera metabolomics in studying the association of the molecular basis of metabolic alterations in breast cancer patients was evaluated [95,96]. Patients with elevated expression of the inositol 1, 4, 5 trisphosphate receptor group showed increased Lac, Ala, lysine, and lipoprotein content, and decreased Glc and Pyr compared to healthy subjects [95]. There was an inverse association between HDL phospholipids and the proliferative index marker (Ki67) in breast cancer patients [96]. The role of serum/plasma metabolic profiles in predicting the outcome to response was investigated [97]. It was shown that metabolic profiles might potentially predict the progression of the disease and overall survival in a subgroup of HER2-positive breast cancer patients on paclitaxel plus lapatinib therapy [97]. In another study using NMR and liquid chromatography-mass spectrometry (LC-MS) using serum metabolic profiling, isoleucine, threonine, Gln (by NMR), and linolenic acid (by LC-MS) were shown as potential biomarkers for response prediction [98]. Stebbing et al. reported the association of metabolic syndrome with adverse outcomes in breast cancer patients by examining serum metabolomics using NMR spectroscopy. High lactate and low alanine combined with high glucose were associated with the progression of the disease [75].

### 3.3. Aspirates

Few studies reported the metabolic profile of fine-needle aspiration cytology (FNAC), fine-needle aspiration biopsy (FNAB), and nipple aspirates samples of breast cancer patients. The ^1^H NMR of FNAC samples showed higher Cho in invasive cancer compared to normal tissues and ductal carcinoma in situ (DCIS) [22]. Metabolic markers such as the Cho to creatine ratio (Cho/Cr) were reported to differentiate malignant from benign samples with 95% sensitivity and a specificity of 96% [20]. A three-stage statistical classification strategy was also developed for the diagnosis and prognosis of breast cancer [23]. Several other metabolites increased, including Cho in FNAC samples of breast cancer compared to benign aspirates and other breast cytopathology [21]. Using NMR and GC-MS, Tredwell et al. identified 38 metabolites, including fatty acids, carbohydrates, amino acids, and organic acids in nipple aspirate. They suggested it can also serve as a source of biomarkers for assessing breast cancer risk and predicting response [30].

### 3.4. Cell-Line Models

The metabolomics of cell lines serves as a valuable model for understanding the molecular mechanism of underlying alterations in breast cancer metabolism and evaluating new therapeutic targets [26,31,95]. Gowda et al. targeted glutaminase using its inhibitor BPTES (bis-2-(5-phenylacetamido-1,3,4-thiadiazol-2-yl) ethyl sulfide) in two breast cancer cell lines, MDA-MB231 and MCF7 [31]. The metabolic profile revealed the association of cancer proliferation with Gln addiction. The inhibition of glutaminase altered glycolysis, Kreb’s cycle, nucleotide, and amino acid metabolism. Metabolic alterations were found to be higher in MCF7 than in MDA-MB231 cell lines [31]. Singh et al. [95] reported a significant decrease in Glc uptake in MCF-7, MDA MB-231, and MCF 10 cells by blocking the inositol 1,4,5 trisphosphate receptor using small interfering RNA (siRNA). The Glc uptake showed more reduction in MDMBA-231 and MCF 7 than in MCF 10 cells [95].

The NMR of breast cancer cell lines was utilized to understand the influence of the tumor microenvironment on lipid and Cho metabolism [55,99]. Cao et al. [100] investigated the effects of silencing two glycerophosphodiesterase genes, GDPD5 and GDPD6, using siRNA on Cho and lipid metabolism in two breast cancer cell lines, MDA-MB-231 and MCF-7. They reported a significant increase in GPC levels, while no change in PC and free Cho levels, silencing both GDPD5 and GDPD6, supported their role as GPC specific regulators [99].

### 3.5. Breast Biomarkers: Living Tissue In Vivo ^1^H, ^31^P, and Hyperpolarized ^13^C MRS

In vivo ^1^H MRS provides a noninvasive measure of metabolites from a localized region from the human breast. Several studies reported a higher water-to-fat (W-F) ratio in malignant breast tissues compared to normal tissues and benign lesions, suggesting its role in monitoring the effect of chemotherapy in breast cancer [100,101,102]. The association of water and fat content with the risk factors of breast cancer was also reported [103]. A lower fat fraction in cancer compared to benign and healthy breast tissues was documented [104]. This parameter showed a 76% sensitivity with a specificity of 74.5% to discriminate cancer from benign lesions [104]. Differences in fatty compositions were noticed in malignant and benign lesions and among various molecular subtypes of breast cancer [105]. The use of the W-F ratio in monitoring the therapeutic response of breast cancer demonstrated a sensitivity of 71% and an accuracy of 79% [101].

**Figure 4:**
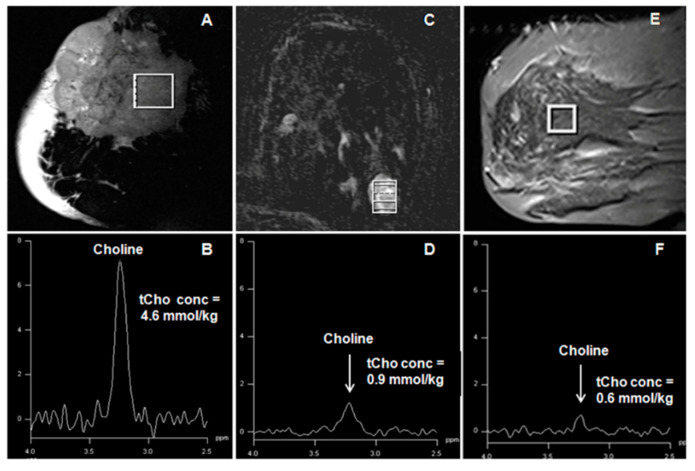
(**A**,**B**): T2-weighted sagittal MR image showing the voxel location from a malignant lesion and the corresponding ^1^H MR spectrum acquired from 20 × 20 × 20 mm^3^ voxel. (**C**,**D**): Dynamic contrast-enhanced axial MR image showing the voxel location from a benign tumor and the corresponding spectrum acquired from 10 × 11 × 15 mm^3^ voxel. (**E**,**F**): T2-weighted sagittal MR image showing the voxel location from normal breast tissue and the corresponding ^1^H MR spectrum acquired from 15 × 15 × 15 mm^3^ voxel (Reproduced with permission from John Wiley & Sons, Inc. (Hoboken, NJ, USA) from Reference [42]).

In the water and lipid suppressed MR spectrum, 1.5T showed a tCho resonance at 3.22 ppm (Figure 4), which served as a noninvasive biomarker in discriminating breast cancer from benign breast diseases [40,41,42,43,44,45,46,47]. A metaanalysis of pooled data including 773 malignant lesions and 452 benign lesions from 19 studies showed the pooled sensitivity and specificity of MRS as 73% and 88%, respectively [45]. ^1^H MRS performed at the higher field, 3T documented increased sensitivity of tCho detection with better resolution [56,57,58]. Recently Clauser et al. reported that a combination of multiparameters such as a signal- to- noise ratio of tCho signal, lipid peak (5.34 ppm), and W-F ratio in multiple regions enhance the diagnostic performance of ^1^H-MRS [59]. Similarly, Thakur et al. reported that the combined use of tCho and W-F ratios might help differentiate different tumor subtypes of cancer and benign lesions and increase the diagnostic usefulness [106]. It was possible to differentiate the infiltrating ductal carcinoma (IDC) and intralobular carcinoma (ILC) lesions with similar tCho levels using the W-F ratio [106]. Both W-F ratio and tCho were demonstrated as a marker of response assessment in patients undergoing neoadjuvant chemotherapy (NACT) [41,101,102,107,108,109,110]. The level of the tCho was found to reduce in LABC patients responding to chemotherapy [41,107,108,109,110].

**Figure 5:**
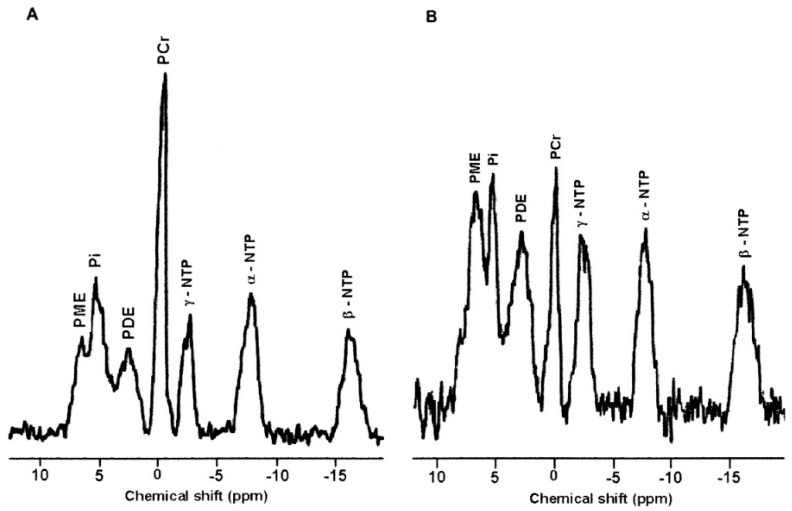
(**A**) ^31^P MR spectrum from the normal breast tissue of a volunteer. NTP- nucleotide triphosphate; PDE-phospho-diesters; PME-phospho-monoesters; PCr-phosphocreatine; Pi-inorganic phosphate. (**B**) ^31^P MR spectrum of a patient suffering from IDC (Reproduced with permission from from Springer from Reference [111]).

In addition, several studies used MR spectroscopic imaging (MRSI) to sample the spectra from multiple voxels and characterize breast cancer heterogeneity with the simultaneous evaluation of multiple lesions [12,108]. ^31^P MRS can also be used to measure PC, and other membrane metabolites to discriminate malignant from benign lesions and normal breast tissue [111,112] (see Figure 5). For example, a recent ^31^P MRS study at 7T showed associations between relative levels of phosphomonoester (PME) and phosphodiester (PDE) with metabolic activity as measured by mitotic count [112].

Hyperpolarized (HP) ^13^C MRS is another emerging clinical tool to probe the aerobic glycolysis or Warburg effect in breast cancer. HP ^13^C MRI uses ^13^C labeled substrates that increase the MRS signal acquired by more than 10,000-fold [48,113,114]. ^13^C-labeled Pyr is the most widely used substrate injected intravenously, and then HP ^13^C-lactate produced from it is measured in real-time using ^13^C MRS [115]. HP ^13^C-lactate labeling revealed the disease aggressiveness of the tumor metabolic phenotype in preclinical studies [116]. Gallagher et al. reported that the Lac/Pyr ratio was significantly correlated with tumor volume, monocarboxylate transporters, and HIF1α expression in breast cancer patients [48]. In a recent study, Woitek et al. [117] reported the use of HP ^13^C MRI in the early prediction of the NACT response and compared it with pharmacokinetic parameters Ktrans and kep derived from DCE-MRI. Reduction in the ^13^C-labeled Lac/Pyr ratio by 34% correctly identified a pathologic responder after one cycle of NACT, while pharmacokinetic modeling using DCE-MRI incorrectly showed poor response to therapy [117].

### 3.6. Breast Biomarkers: Dynamic Contrast-Enhanced MRI (DCE-MRI)

**Figure 6:**
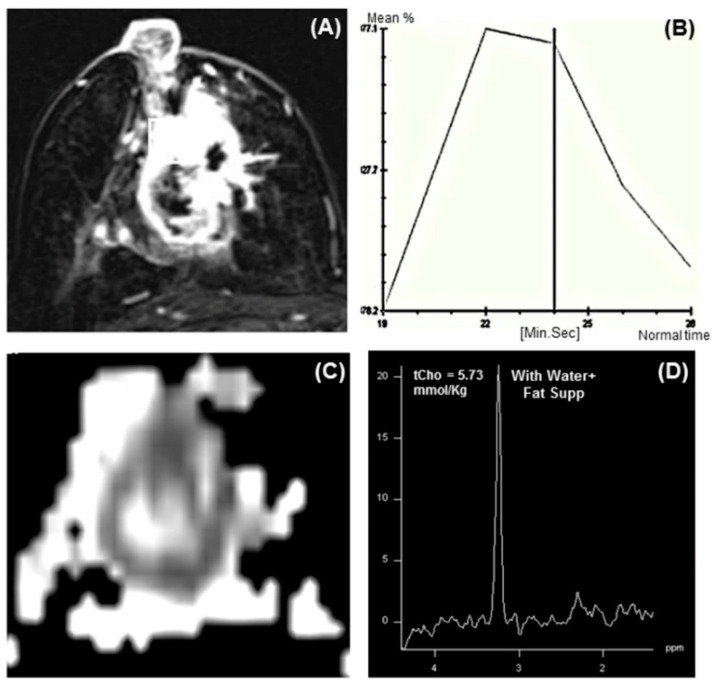
(**A**) Representative DCE-MR image of a 56-year-old locally advanced breast cancer patient suffering from IDC, and (**B**) the corresponding type III curve obtained from the ROI positioned on the lesion. (**C**) shows the ADC map while (**D**) is the in vivo ^1^H MR spectrum of the same patient (Reproduced with permission from Elsevier from Reference [119]).

DCE-MRI measures tumor vascularity and blood perfusion, which has significant potential in investigating the biological characteristics of tumors [3,4,12,118]. It relies on administering an intravenous gadolinium-based contrast agent [3,4,12,118]. The growth of cancer requires the generation of new blood vessels for a sufficient supply of nutrients. These new vessels are leaky, and gadolinium contrast agents can extravasate from them and accumulate in the tumor stroma. Basic DCE-MRI protocol consists of one pre-contrast T1-weighted image followed by a sequence of post-contrast T1-weighted images, which are then used to determine the kinetics of contrast accumulation in a tumor (Figure 6). Since vessels are leaky in the tumors, it leads to rapid washouts. DCE-MRI enables the characterization of lesion morphology and contrast kinetics using pharmacokinetic modeling [3,4,12]. The pharmacokinetic modeling of various parameters such as Ktrans and Ve allows the measurement of tissue perfusion.

Breast MRI has become the most sensitive tool for detecting breast cancer. The use of multiparametric (mpMRI) protocols that combine the information from various MR techniques can increase the specificity of breast MRI [3,4,12,120]. DCE-MRI is recommended as a breast cancer screening modality for women with increased risk [3,4]. DCE-MRI outperforms conventional mammography in early breast cancer detection and provides a preoperative assessment of lesions [3,4].

Abbreviated MRI protocols were recently introduced to enable their wider use and to reduce cost. They showed equal performance to extended multiparametric protocols [120,121]. Ultrafast DCE-MRI is another recently developed method that enables high spatial and temporal resolution. Various acceleration methods, such as parallel imaging and compressed sensing, are used in ultrafast DCE-MRI. Moreover, when used in the very early phase, it can generate contrast kinetics reflecting inflow effects. In recent years, the utility of ultrafast DCE-MRI-derived parameters was demonstrated in the characterization of breast cancer aggressiveness and tumor subtypes [122].

### 3.7. Breast Biomarkers: Perfusion-Weighted Imaging

The characteristics of new vessels generated for the proliferation of cancer are different from normal vessels and are larger in diameter, have more permeability, and lack contractile properties [5]. Therefore, specific MRI sequences are developed, both contrast-based and noncontrast, to measure tissue perfusion.

Contrast-based first-pass perfusion imaging provides a measure of blood volume, which is directly related to tumor microvascular perfusion. Several studies documented that the tumors had increased blood flow and volume compared to the normal breast tissue [123,124,125,126,127,128]. Park et al. [129] investigated the association between MRI perfusion parameters and clinical and pathologic variables in patients with TN breast cancer. The pharmacokinetic analysis of DCE-MRI perfusion parameters was based on the extended Tofts model [130]. The higher values of Ve and peak enhancement at pre-therapy were associated with worse survival [129].

It was reported that the tumors with higher stroma showed significantly higher Ve [131]. Several other studies confirmed the correlation between tumor cellularity and poor prognosis with Ve [132,133]. The patients with an elevated intratumor stroma had a shorter relapse-free period and overall survival in numerous studies, indicating it as a prognostic factor [134,135,136], specifically in TN breast cancer patients [134,135,136]. Liu et al. [137] investigated the association of perfusion parameters obtained through preoperative DCE-MRI with Ki-67, HER-2, ER, PR, cytokeratin 5/6 (CK5/6), and epidermal growth factor receptor (EGFR). The Ki-67 showed a significant positive correlation with the rate constants, Ktrans, and kep. The value of Ve was significantly different between tumors positive and negative for CK5/6. HER-2-enriched tumors showed higher kep than luminal A tumors.

The measurement of perfusion parameters without using contrast media was developed and evaluated [123,124,138]. One such technique is arterial spin labeling (ASL) to assess tissue perfusion. ASL could be a promising noncontrast-enhanced alternative for DCE-MRI, providing information on perfusion and vascularity. The advantages of ASL are that it does not require contrast media and is not sensitive to vessel wall permeability. In ASL, the magnetic labeling of endogenous blood is used to achieve perfusion contrast [138]. In this technique, two images, a magnetically labeled and a control image, are acquired alternatingly. An ASL image is obtained by subtracting the label and control images. On subtraction, static tissue signals cancel out, only labeled blood signal remains. The commonly used ASL techniques for body and brain applications are pseudo-continuous ASL (pCASL), [139,140] and flow-sensitive alternating inversion recovery (FAIR) [141,142], which are spatially selective. In these techniques, blood labeling occurs in the tissue’s feeding arteries [138], leading to a transit delay between the location of labeling and the arrival of this blood in the tissue. Another recently developed technique is velocity-selective ASL, in which blood is labeled based on the velocity of flow instead of the location [143]. The blood above a specific cutoff value of velocity is labeled, and the cutoff can be chosen to eliminate the transit delay. Velocity-selective ASL was evaluated for its technical feasibility for bilateral imaging in breast cancer. The study reported that the morphology of the lesions seen on velocity-selective ASL corresponded to early phase ultrafast DCE images [143].

### 3.8. Breast Biomarkers: Diffusion-Weighted Imaging (DWI)

DWI is a promising technique that measures the diffusion of water molecules in vivo and can be used to probe microscopic tissue organization [7,8,9,10,11,12,13,14]. The motion of water molecules is random in pure water. However, this motion is restricted in tissues due to hindrances in intracellular and extracellular compartmentalization. Thus, the apparent diffusion coefficient (ADC) measured using DWI reflects tissue characteristics such as cellularity, microstructure, and membrane integrity. Multiple studies demonstrated that malignant breast lesions showed decreased ADC compared to benign lesions and normal breast parenchyma [7,8,9,10,11,12,13,14]. Decreased ADC in malignant lesions is primarily attributed to increased cell density due to the uncontrolled proliferation of cancer cells [7,8,9,10,11,12,13,14]. The advantage of DWI is that it has a short scan time and is a noncontrast technique [12]. In addition, the higher accuracy of DWI was reported in a study of asymptomatic women for detecting malignancy compared to screening mammography [144].

ADC measures discriminated benign and malignant breast lesions and complemented DCE-MRI for increasing the specificity of breast MRI [7,9,12,14]. A metaanalysis including 964 (349 benign, 615 malignant) breast lesions combined from 13 studies reported that the pooled sensitivity for detecting the malignancy was 84%, and the specificity was 79% for DWI [13]. The role of ADC in discriminating malignant and benign lesions and the association with molecular biomarkers such as ER, PR, and HER2 was investigated [8]. ADC was found to be useful in identifying malignancy in breast cancer patients with indeterminate DCE curve findings [119]. Further, TN showed significantly higher ADC than non-triple negative (nTN), ER+, PR+ cancers, indicating ADC association with the molecular biomarkers [8]. Richard et al. reported that pretreatment tumor ADC values varied between tumor subtypes and predicted a pathological response in TN tumors in LABC patients undergoing NACT [145].

**Figure 7:**
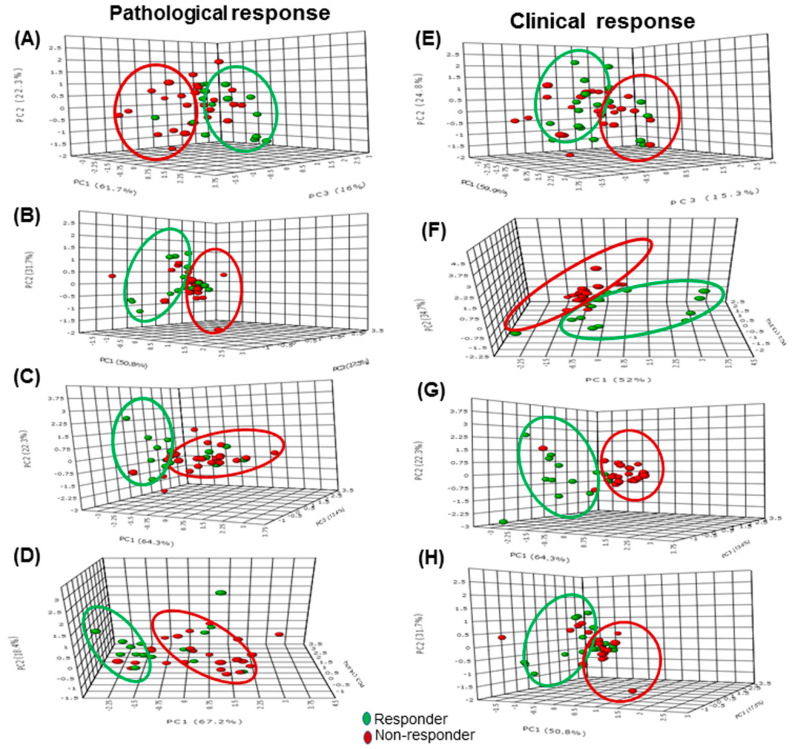
The 3-D score plot (PC1-PC3) of PCA analysis of multi-parametric data (volume, ADC, and tCho) in pathological responders and nonresponders at Tp0 (**A**) after Tp1 (**B**), Tp2 (**C**), and Tp3 (**D**), while (**E**–**H**) show the 3-D score plot for clinical response. (Reproduced with permission from Reference [110]: Sharma, U.; Agarwal, K.; Sah, R.G.; Parshad, R.; Seenu, V.; Mathur, S.; Gupta, S.D.; Jagannathan, N.R. Can a multi-parametric MR based approach improve the predictive value of pathological and clinical therapeutic response in breast cancer patients? *Front. Oncol*. 2018, *8*, 319. doi: 10.3389/fonc.2018.00319).

Another major application of DWI in breast cancer management is monitoring the response to therapy [7,9,12,13,111,145,146]. It was reported that patients responding to chemotherapy and radiotherapy showed increased ADC values. In addition, it was reported that changes in ADC can predict tumor response as early as the first cycle of NACT, earlier than changes in tumor size detected by conventional MRI [111,146]. We recently investigated the potential of a multi-parametric MR approach based on the measurement of tCho, ADC, and tumor volume in predicting the pathological (pR) and clinical response(cR) to NACT for patients with LABC (Figure 7). After the third cycle of NACT, the MR volume showed the highest sensitivity (96.2% for cR, and 83.3% for pR) to detect responders while specificity was highest for ADC (100% for cR, and 76.5% for pR) than the combined use of all parameters [111].

Advanced approaches such as intravoxel incoherent motion (IVIM) modeling are used to characterize breast malignancy [147,148,149]. Besides diffusion, these methods also provide information on tissue perfusion [147,148,149]. Additionally, diffusion kurtosis modeling [148,150] and diffusion tensor imaging (DTI) [151,152] are also being investigated to characterize the directionality of water diffusion and understand tissue complexity.

### 3.9. Breast Biomarkers: MR Elastography (MRE)

In breast cancer, the reactive proliferation of connective tissue leads to the accumulation of a dense layer of fibroblasts around malignant epithelial cells [153]. It results in hardening or change in the stiffness of the breast tissue, which is diagnosed by palpation. MRE is a noninvasive imaging technique to measure the stiffness or elasticity of tissues and uses low-frequency vibrations to quantitatively measure the visco-elastic properties of tissue during malignant transformation [15,16,17,154]. The stiffness of tumors is related to tumor development, metastasis, invasion, and resistance to chemoradiotherapy [154].

In MRE, acoustic waves are applied to the tissue (range, 100 to 1000 Hz), and the propagation of these waves is imaged using motion-sensitive MRI sequences. The acquisition timing can be varied to observe the waves at various time points. The wavelength can be estimated by processing the wave images, from which the shear modulus value and shear modulus map can be produced. For example, MRE in healthy volunteers and patients with breast cancer with 100 Hz shear wave showed that the stiffness of breast cancer tissue was four times higher than that of normal breast tissue [155]. Similarly, many studies confirmed that the elasticity of breast cancer was increased compared to normal breast tissue and benign lesions [156,157]. For example, Balleyguier et al. reported that the sensitivity and specificity value of MRE for breast cancer diagnosis was 79% and 90%, respectively, in breast cancer patients with high BIRADS scores [158]. They also reported a higher value of AUC (0.92) for MRE compared with 0.84 for MRI alone [158]. Similarly, studies reported an increase in the specificity of MRI on the addition of MRE while maintaining a high sensitivity [17,159].

### 3.10. Radiomics

Radiomics is a rapidly evolving field that applies artificial intelligence algorithms to digitally decode medical images, enabling the comprehensive characterization of tumors [160,161,162]. The radiomics approach is based on the concept that aims to obtain quantifiable data from images that are not analyzed conventionally by radiologists [161]. It includes the extraction of quantitative properties or features, including descriptors of tumor shape, size, intensity, and texture which are then utilized as inputs in machine learning algorithms providing outputs concerning disease classification and outcome predictions [161,162]. The radiomics approach is expected to have significant applications in addressing several clinical challenges such as cancer detection, the assessment of therapy response, disease recurrence, prognosis, and disease progression. Radiomics is based on the hypothesis that the extracted quantitative features are the phenotypic manifestation of underlying genetic and molecular alterations occurring with malignant transformation at genetic and molecular levels. The radiomics approach is detailed in the literature [160,161,162]. Several studies investigated the potential of radiomics in differentiating benign breast lesions from malignant tumors [162]. These studies demonstrated that the addition of radiomics can improve the diagnostic accuracy of conventional breast imaging [162]. For example, an MRI-based radiomics retrospective study on 26 benign and 98 malignant patients reported that entropy of malignant tumors was significantly higher on DCE-MRI and ADC maps than benign lesions [163].

Furthermore, the radiomic feature maps (RFM) demonstrated significantly different RFM curves for malignant and benign lesions using DCE-MRI and DWI, indicating the correlation of radiomics features with the vascularity and heterogeneity of tumors, respectively. Another study based on DCE-MRI (264 benign lesions and 390 luminal A breast cancers) extracted 38 quantitative features to differentiate luminal breast cancers from benign breast lesions [164]. The inclusion of radiomic features led to a significant improvement in the ability to differentiate between luminal A breast cancers and benign lesions, compared to size alone [164].

The radiomics approach was also been used to evaluate its prognostic potential in predicting sentinel lymph node, axillary lymph node, or sentinel lymph node metastases [165,166] in breast cancer patients. A high correlation was documented between radiomics features extracted from DWI with sentinel lymph node metastases [165]. Recent studies evaluated the radiomics approach to predict the expression of proliferation marker Ki67 [167,168]. Tagliafico et al. [168] reported a prospective study on 70 women diagnosed with breast cancer for the potential use of the evaluation of radiomics features in the prediction of Ki67 expression. An AUC of up to 0.698 was obtained using five features. A significant correlation between Ki-67 and 34 features was reported.

## 4. Summary, Outlook, and Future Directions

This review briefly presented the potential of MRI and MRS-based methods in understanding breast cancer biology and the role of various MR biomarkers in disease diagnosis, prediction, screening, therapeutic monitoring, and tumor recurrence. The in vitro high-resolution NMR studies of tissue extracts, nodes, serum, and blood plasma samples detected many metabolites in breast cancer patients. Altered levels of several metabolites including Glc, Lac, membrane metabolites such as tCho, GPC, and amino acids such as Ala, Glu, Gln, Lys, His, Gly, Ser, and Tau illustrated changes in various metabolic pathways and regulatory mechanisms. Further, these metabolites were potential candidates to serve as diagnostic and prognostic biomarkers in breast cancer management.

The metabolic heterogeneity of tumors was also associated with the molecular heterogeneity of tumors; however, there is a need for the comprehensive metabolic characterization of the heterogeneity of breast cancer lesions. MRI and MRS are currently being used as adjunct methodologies to mammography, histology, and other techniques. MRI offers complementary information on tumor cellularity, perfusion, and stiffness. In recent years, MRI emerged as an important tool for evaluating the population of women at high risk. Many studies demonstrated the use of MRI in detecting mammographically occult cancers. However, breast MRS is still not routinely performed because it is technically challenging. The sensitivity of MRS is also limited by various technical factors. However, recent advances in technological developments, such as increasing the design and sensitivity of breast coils and high-field MR systems, have the potential to improve the accuracy of breast MRS. Though MRI and MRS techniques revealed a large number of biomarkers as potential candidates, to date, these are limited to research laboratories due to several reasons such as technical challenges and higher costs of procedures, nonavailability of equipment, etc. There is a need to develop these approaches with greater reproducibility so that these markers can be used to provide personalized health care in clinics.

There is a need to characterize various histological types of breast cancer using MR approaches for a comprehensive understanding of breast cancer heterogeneity. This can help to increase the diagnostic ability of these methods. Further, there is a need for easy and automated acquisition and post-processing algorithms to visualize and quantify tCho in small-sized tumors. Future research should focus on cutting down the cost of MR procedures for wider applications. Additionally, there is a need for multi-center studies on the application of MRI and MRS approaches to integrate them into clinical settings. There is also a need to evaluate the potential of NMR spectroscopy of biofluids in women with hereditary risk. This is a potential area of further research that can help in stratifying women with high-risk cancer and providing an early indication of the vulnerable population. It is also essential to perform systematically designed metabolomics studies to discover robust biomarkers for the diagnosis and the prognosis of the disease. The results of metabolomics approaches should be translated into developing simplistic methods which could easily be implemented in clinical settings with affordable cost implications. Recent methods such as MR elastography require extensive multi-center investigations. Radiomics applications should be extensively explored, and there is a need to enhance the understanding among radiologists about the basic concepts, development of standardized and reproducible algorithms, and data sharing for clinical applications.

## Figures and Tables

**Table 1:** Comparison of in vitro, ex vivo, and in vivo magnetic resonance spectroscopy (MRS) and MRI techniques.

Characteristics	Magnetic Resonance Spectroscopy			Magnetic Resonance Imaging
	In Vitro	Ex Vivo	In Vivo	
Information	Biochemical composition (metabolite detection)	Biochemical composition (metabolite detection)	Biochemical composition (metabolite detection)	Anatomic (structure and morphology), functional
Sample/Subject	Tissue extract, biofluids, cell lines, aspirates	Excised tissues/biopsies	Living humans/organisms	Living humans/organisms
Equipment	NMR Spectrometer	NMR Spectrometer with accessories for HRMAS	Human MRI Scanner	Human MRI Scanner
Field Strength	High field strength 9.4 T–21.1 T	High field strength 9.4 T–18.8 T	1.5 T–7 T	1.5 T–7 T
Nuclei of interest	^1^H, ^13^C, ^31^P, ^23^Na, ^19^F	^1^H, ^13^C	^1^H, ^31^P, ^23^Na, ^19^F ^13^C- hyperpolarized	^1^H from fat and water
Data	1D/2D spectra	1D/2D spectra	SVS 1D, SVS-2D, CSI (MRSI)	Conventional T1, T2-weighted, DCE-MRI, Diffusion-weighted, Perfusion weighted, MR Elastography, fMRI
Advantages	High sensitivity and resolution, detection of a large number of metabolites, easy quantification, easy experimentation	High sensitivity and resolution, detection of a large number of metabolites, quantification not that easy, special experimentation	Organ-specific metabolite composition, and longitudinal studies.	Organ-specific structural and functional studies, longitudinal studies possible.
Limitations	Tissue excision is invasive	Tissue excision is invasive	Low sensitivity and resolution, detection of a small number of metabolites, Claustrophobia of patients	Claustrophobia of patients, contrast required in some studies
Reproducibility	Lesser than in vivo	Lesser than in vivo	High	High

Abbreviations Used: 1D—one-dimensional spectrum; 2D—two-dimensional spectrum; HRMAS—high-resolution magic angle spinning; SVS—single voxel spectroscopy; CSI—chemical shift imaging; DCE-MRI—dynamic contrast-enhanced MRI.
